# Imaging of Early Response to Predict Prognosis in the First-Line Management of Follicular Non-Hodgkin Lymphoma with Iodine-131-Rituximab Radioimmunotherapy

**DOI:** 10.3390/diagnostics7020026

**Published:** 2017-05-12

**Authors:** Murali Kesavan, Jan Boucek, William MacDonald, Andrew McQuillan, J. Harvey Turner

**Affiliations:** Departments of Haematology and Nuclear Medicine, The University of Western Australia, School of Medicine, Crawley 6009, Western Australia, Australia; jan.boucek@health.wa.gov.au (J.B.); William.MacDonald@health.wa.gov.au (W.M.); Andrew.McQuillan@health.wa.go.au (A.M.); John.Turner@health.wa.gov.au (J.H.T.)

**Keywords:** follicular lymphoma, non-Hodgkin lymphoma, outcome prediction, prognosis, radioimmunotherapy and PET imaging

## Abstract

The purpose of this study was to evaluate prediction of prognosis after first-line radioimmunotherapy (RIT) of advanced follicular non-Hodgkin lymphoma (FL), by imaging with fluorine-18-fluorodeoxyglucose positron emission tomography with computed tomography (^18^F-FDG-PET/CT) three months after induction treatment by Iodine-131-rituximab (^131^I-rituximab). Objective response was determined using the Deauville 5-point scale in 68 prospective clinical trial patients. Baseline ^18^F-FDG-PET/CT studies were used to calculate total-metabolic-tumor-volume (TMTV). Non-imaging studies included the Follicular lymphoma international prognostic index (FLIPI) and absolute baseline monocyte and lymphocyte counts. Patients were monitored for over ten years (median follow-up 59 months), and no patient was lost to follow-up. Complete response (CR) of 88% predicted excellent prognosis with median time-to-next-treatment (TTNT) not yet reached. Those patients (12%) who failed to achieve CR (Deauville ≤ 3) on ^18^F-FDG-PET/CT at three months had significantly poorer outcomes (*p* < 0.0001) with a median TTNT of 41 months. Requirement for re-treatment was predicted by FLIPI and absolute baseline monocyte count but not lymphocyte count. The TTNT was accurately predicted by ^18^F-FDG-PET/CT Deauville response at three months following first-line therapy of FL with RIT. Early response demonstrated by imaging does, therefore, foretell prognosis in the individual FL patients.

## 1. Introduction

Follicular lymphoma (FL) constitutes over 20% of all non-Hodgkin lymphoma (NHL) [1]. When treated with rituximab alone, only 12% of patients required a new treatment within three years, compared with over half of the patients left untreated on a watch-and-wait protocol [2]. Combination of chemotherapy with rituximab has led to a remarkable prolongation of remission over the past decade; however, 20% of FL patients treated with immunochemotherapy have disease progression within two years and a five-year overall survival (OS) of only 50% [3]. Existing prognostic indices at the time of induction therapy do not reliably identify these patients who are destined to relapse. The wide spectrum of clinical, biological and genetic heterogeneity of FL has confounded accurate prediction of the quality, degree, and duration of response to first-line treatment in any given individual patient in routine haemato-oncologic practice. Patients with a poor outlook are not easily defined by pre-treatment prognostic indices—e.g., Follicular Lymphoma International Prognostic Index (FLIPI and FLIPI2) [3] or conventional response assessment with 1999 International Workshop Criteria [4].

Assessment with the International Workshop Criteria (IWC) imaging-based index involves complex calculations on contrast-enhanced computed tomography (CT) of lymph node dimensions to distinguish the grade of response, and, not only is it impractical for everyday clinical practice, its use has been superseded by positron emission tomography (PET) [4]. Such impracticality also compromises the usefulness of the recently augmented m7-FLIPI, which integrates the mutation status of seven genes into a clinicogenetic risk model for prognostication of patients receiving induction immunochemotherapy for FL [5]. Nevertheless, FLIPI is a validated instrument for predicting survival outcomes in advanced FL treated front-line with rituximab combined with cyclophosphamide, doxorubicin, vincristine and prednisone (R-CHOP) [6].

The blood parameters not included in the FLIPI index have been independently evaluated for prognostication, and each has major shortcomings. Whilst the degree of bone marrow infiltration by tumor on biopsies at diagnosis is a general risk factor, its assessment poses several problems in practice. Due to the sporadic nature of marrow invasion by tumor, the sampling error is high. In addition, even given an adequate representative tissue biopsy, the pathological examination is subjective. The ability of pathologists to consistently divide patients into grades 1, 2 or 3 was shown to be only 60–70% reliable [1]. This does not yield much confidence that they will be able to subdivide grade 3 FL consistently. Post induction and follow-up bone marrow biopsies are not usually performed and the very low incidence of detectable residual involvement suggests little additional prognostic value [4].

A non-invasive simple blood test measuring the absolute monocyte and lymphocyte count may reflect the complex relationship of FL with infiltrating immune cells and has been shown, in the case of monocytes, to predict survival outcome [7].

The standard diagnostic tool in lymphoma is fluorine-18-fluorodeoxyglucose positron emission tomography with CT (^18^F-FDG-PET/CT) as adopted at the first international workshop on PET in lymphoma in Deauville, France, 2009 [8]. The ^18^F-FDG-PET/CT study, performed at three months after induction therapy of FL, is reported according to the Deauville 5-point criteria and allows inter-trial comparisons. This evaluation is eminently practical and is now available to haemato-oncology clinicians worldwide as the current standard of care [9].

The efficacy of ^18^F-FDG-PET/CT for prognostication after R-CHOP induction therapy of FL, using comparison of baseline and post treatment images, was demonstrated in a prospective study where the two-year progression free survival (PFS) was 87% versus 51% for PET negative versus PET positive patients respectively on the follow-up scan [10].

In our prospective clinical trial of first-line radioimmunotherapy (RIT) of advanced FL using ^131^I-rituximab [11], we applied all the standard prognostic indices and here report their predictive value in our 10-year follow-up study. In addition we have incorporated the estimation of total-metabolic-tumor-volume (TMTV) on the ^18^F-FDG-PET/CT, which has recently been claimed to predict outcome in FL [3].

## 2. Patients and Method

We reviewed the 68 patients who received first line ^131^I-rituximab RIT for newly diagnosed grade 1 or 2 CD20-positive follicular lymphoma as part of the original prospective phase II INITIAL study commenced on 15 February 2007 (Australian Clinical Trial Registry Notification No. 12607000153415) [11]. Long-term outcome data are reported based on clinical disease status as of 7 of January 2017.

Disease stage at baseline was determined by ^18^F-FDG-PET/CT and bone marrow biopsy. Standard eligibility criteria were applied; baseline entry myeloid function required platelets >70 × 109/L, neutrophils >1 × 109/L and hemoglobin >100 g/L. All patients met Groupe d’Etude des Lymphomes Folliculaires (GELF) criteria for treatment [12]. The median age of study population was 60 years, given that overall survival (OS) in FL is greater than 10 years and that relapse may not require therapy, we used time-to-next-treatment (TTNT), rather than progression free survival (PFS), as the most clinically relevant measure of outcome.

To obviate the effect of possible tumor lysis response to the initial dose of rituximab, the second rituximab administration was used to calculate individualized patient dosimetry of iodine-131 radiolabeled antibody. Individual dosimetry was performed using tracer 250 MBq ^131^I-rituximab whole body SPECT/CT imaging during the week following this second administration of rituximab. A prescribed radiation absorbed dose to the whole body was fixed at 0.75 Gy and each patient received an individual administered activity (GBq) for therapy following the week 3 administration of rituximab. All patients received true radioimmunotherapy, such that each patient had four doses of 375 mg/m^2^ of non-radioactive rituximab immunotherapy plus 0.75 Gy of iodine-131-rituximab (15 mg) radionuclide therapy. Patients were monitored with weekly blood counts during the first eight weeks and reviewed at regular intervals thereafter.

Patients who achieved a treatment response received standard maintenance treatment comprising four 375 mg/m^2^ doses of non-radiolabeled rituximab each administered every three months for 12 months.

Deauville response criteria were assessed by ^18^F-FDG PET/CT scans performed three months after RIT. In particular, achievement of Deauville score of 3 or less is deemed a CR [13]. All ^18^F-FDG-PET/CT studies were reviewed by the study nuclear physician (WBGM).

Physicist of record (JB) calculated the TMTV using the published 41% maximum standardized uptake value thresholding method [3] in conjunction with the ^18^F-FDG-PET/CT reports of the physician of record (WBGM). Only tumoral lesions were evaluated, lesion-by-lesion, each with its individualized region of interest (ROI). This methodology has been published and is now referenced [14]. Spleen was only considered if there was focal uptake or diffuse uptake higher that 150% of the liver background [3]. Only studies with full PET/CT trans axial acquisitions were used and all of these original images were attenuation corrected. Eight of our 68 patients were scanned with PET only technology and were not included in this study of TMTV.

Specific prognostic factors analyzed included age greater than 60 years, gender, baseline lymphocyte count ≥1.2 × 10^6^/L [15,16] (lower limit or normal for our laboratory), baseline monocyte count ≥0.8 × 10^6^/L [17,18] (upper limit of normal for our laboratory), follicular lymphoma international prognostic (FLIPI1 and FLIPI2) [19,20,21,22] score at diagnosis, presence of disease bulk (defined as >7 cm), baseline total body metabolic tumor volume[3] and Deauville response to therapy [23].

Time-to-next-treatment and survival comparisons were made using Kaplan–Meier methodology. Risk ratios and survival curves were compared using the log-rank test, except in the presence of non-proportional hazards (when early crossing of the curves was identified), where the Gehan–Breslow–Wilcoxon test was applied [24]. All data was analyzed using SPSS v18.0 (IBM Corp., Armonk, NY, USA).

## 3. Results

Of the 68 evaluable patients none have been lost to follow-up. The median age at enrollment was 60 years (37 females and 31 males, age range 31–89 years). The median duration of follow-up at the cut-off date was 59 months. Fifty-one patients (75%) had stage III/IV disease with 27 patients (40%) having a FLIPI of III/IV. Baseline and follow-up TMTV was evaluable in 60 patients. The median baseline TMTV in these patients was 116 cm^3^ with five patients (8%) having high-tumor burden, defined as TMTV greater than 510 cm^3^. Full baseline characteristics are outlined in Table 1.

### 3.1. Response

Deauville criteria were fulfilled for complete response (Deauville ≤ 3) based on ^18^F-FDG PET/CT at three months follow-up in 60 patients (88%). Of the remaining eight patients, seven (10%) had partial response (PR, Deauville 4 or 5) and one patient (1.5%) had primary progressive disease and did not have a three-month ^18^F-FDG-PET/CT assessment due to rapid progress.

**Figure 1:**
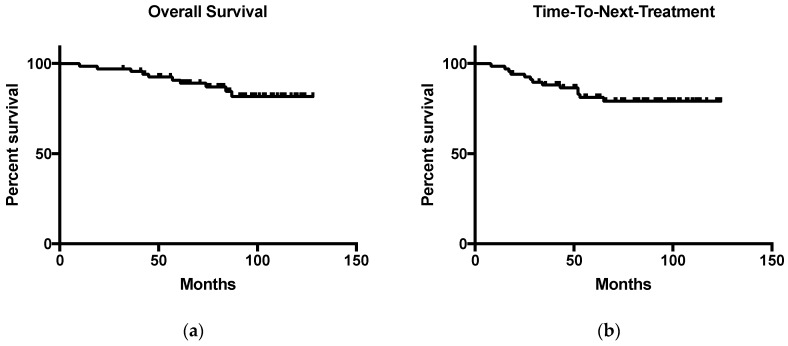
^131^I-rituximab therapy outcomes. (**a**) Kaplan-Meier plot of overall survival (OS), median OS is yet to be reached; (**b**) Kaplan-Meier plot of time-to-next-treatment (TTNT), median TTNT is yet to be reached.

Of the 67 patients (99%) achieving a response CR/PR, 15 (22%) have experienced progression or relapse by end of follow-up (median 59 months). Twelve patients (18%) required retreatment. Eighty-six percent (58 of 67) of initial responders remain alive. Ten (15%) deaths have occurred; three due to disease related causes (progressive/transformed disease), four due to non-lymphoma related causes (two from complications of dementia, one from pneumonia without prior evidence of disease recurrence and one from complications of Parkinson’s disease) and two due to other malignancies (melanoma and brain tumor). The median OS has not been reached (Figure 1a).

In total, 16 patients (23%) have experienced progression/relapse with 13 patients (19%) requiring retreatment (eight complete responders, four partial response and the one patient with primary progressive disease). Twelve of these patients had required re-treatment within five years of completing therapy. The median overall TTNT has not been reached (Figure 1b).

### 3.2. Prognostic Factors

**Figure 2:**
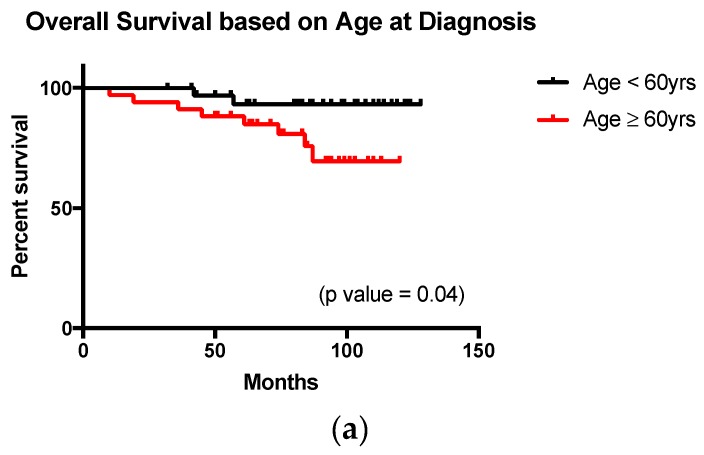

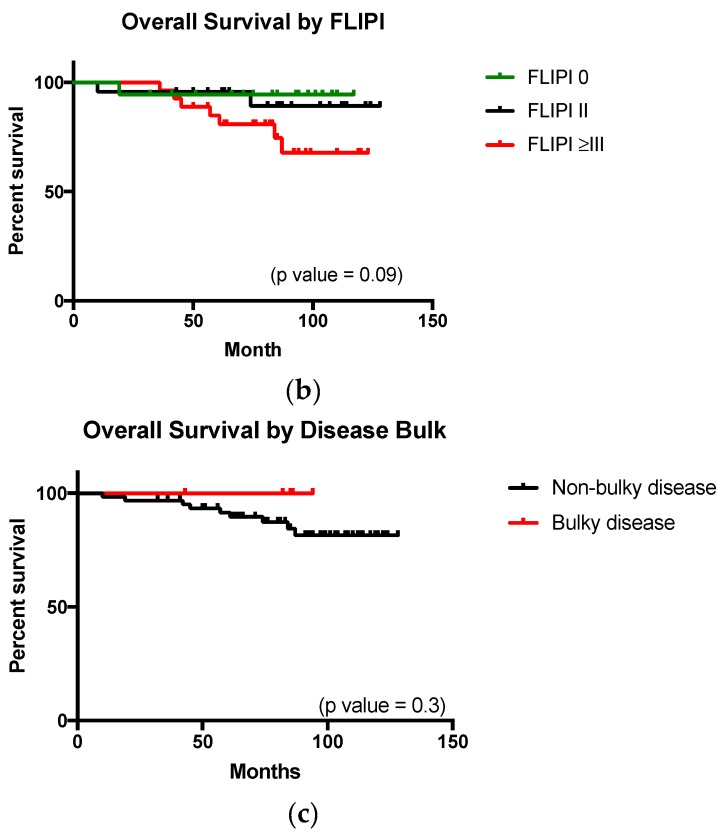
Survival analysis. (**a**) Patients aged >60 years at diagnosis had significantly reduced survival; (**b**) Demonstrates inferior survival for patients with a baseline FLIPI ≥3, however this result is not statistically significant; (**c**) Baseline disease bulk (largest nodal mass >7 cm in diameter) was not associated with a reduced overall survival. However this result is likely due to the limited number of patients with baseline disease bulk enrolled in the study.

With respect to OS, the most significant prognostic factor identified was that of age, with age greater than 60 years associated with a relative risk (RR) of 4.3 (*p*-value 0.04) (Figure 2a). Although a FLIPI >3 and presence of disease bulk >7 cm conferred an RR of 3.5 (*p*-value 0.09) and 3 (*p*-value 0.3), respectively. Neither result was statistically significant (Figure 2b,c). Gender, lymphocytosis, monocytosis >0.8 × 10^6^/L or high tumor burden based on TMTV >510 cm^3^ had no impact upon overall survival (findings summarized in Table 2).

**Figure 3:**
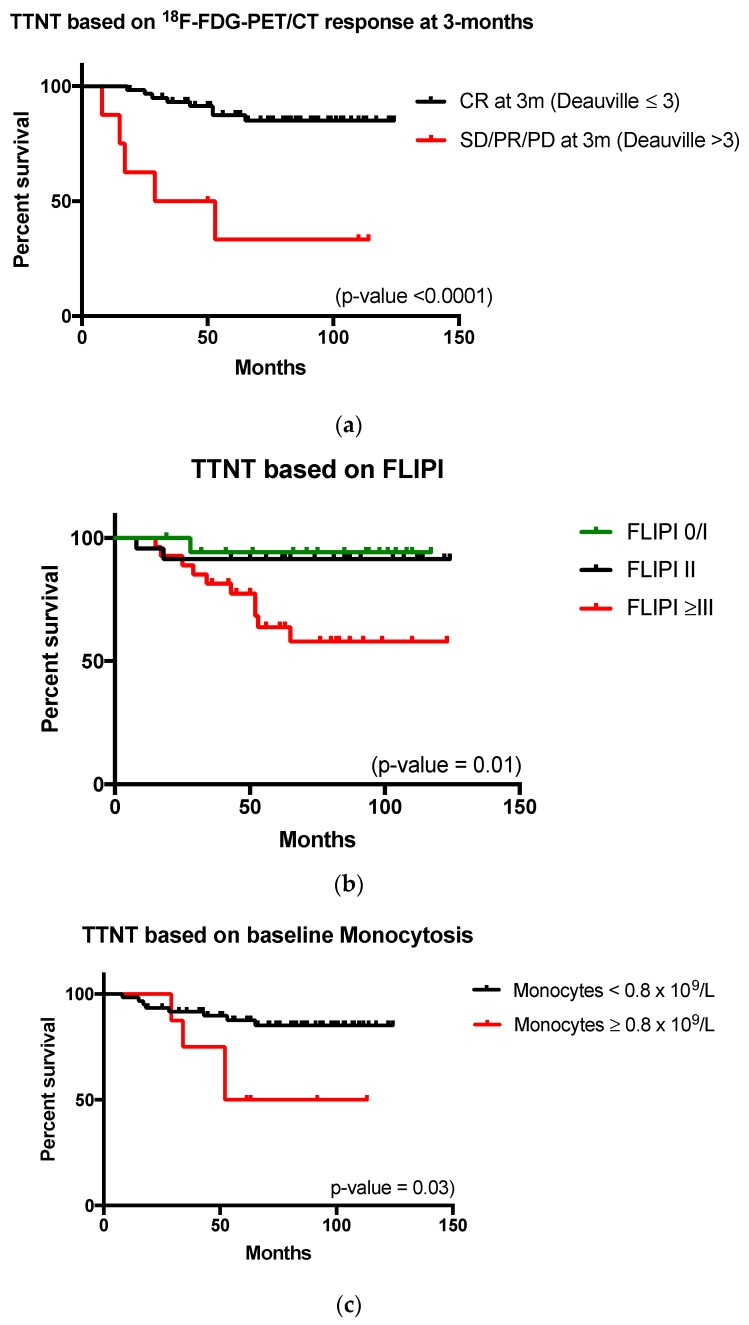
Time-to-next-treatment (TTNT) by imaging and non-imaging parameters. CR: complete response PR: partial response SD: stable disease PD: progressive disease. FLIPI: Follicular Lymphoma International Prognostic Index. (**a**) Patients failing to achieve a complete remission were more likely to experience relapse and require re-treatment. The median TTNT was 41 months for those failing to achieve a complete response by Deauville 5-point scale at the 3-month post treatment imaging study; (**b**) Patients with a baseline FLIPI 3 were at significantly higher risk or relapse and subsequent re-treatment; (**c**) Baseline monocytosis (as per the local laboratory upper limit of normal value of 0.8 × 10^6^/L) was also able to identify patients at risk of requiring re-treatment.

**Figure 4:**
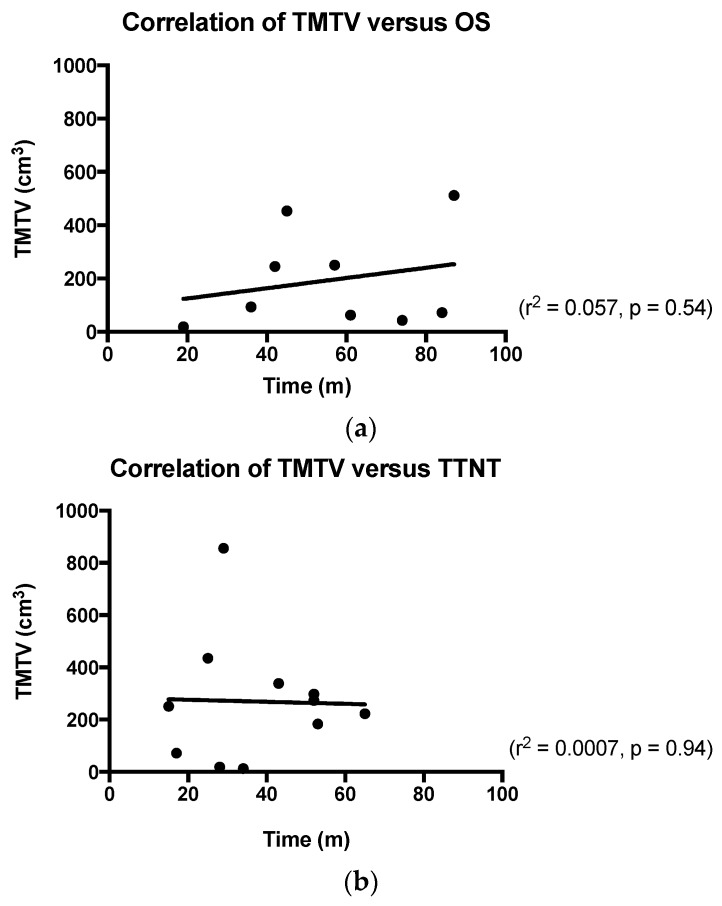
Total-metabolic-tumor-volume analysis. TMTV: Total-metabolic-tumor-volume; OS: Overall survival; TTNT: Time-to-next-treatment. (**a**) Linear regression analysis of baseline TMTV versus overall survival failed to identify a significant correlation; (**b**) Similarly, linear regression analysis of TMT versus TTNT also failed to identify a significant correlation.

With respect to TTNT, the most significant prognostic factor was a failure to achieve CR on the three-month ^18^F-FDG-PET/CT study. The relative risk (RR) of requirement for retreatment in patients who failed to achieve such a CR was 7.4 (*p*-value < 0.0001). The positive predictive value and negative predictive value of ^18^F-FDG-PET/CT for five-year treatment free survival was 88% and 62%, respectively (95% CI 0.78–0.94). The median TTNT for patients with a Deauville score >3 was 41 months and not yet reached in the cohort who achieved a Deauville score ≤3 (Figure 3). Baseline monocytosis >0.8 × 10^6^/L and FLIPI >3 were also noted to be significant, conferring an RR of 9.2 (*p*-value 0.03) and 5.6 (*p*-value 0.01), respectively (Figure 3). In comparison to the survival analysis, age greater than 60 years did not greatly contribute to the risk of relapse and subsequent need for re-treatment. Gender, lymphocytosis >1.2 × 10^6^/L, disease bulk and high tumor burden based on TMTV >510 cm^3^ were not significant (Full findings summarized in Table 3). Moreover, linear regression analysis of baseline TMTV versus OS/TTNT failed to identify a statistically significant correlation between the variables (*r*^2^ = 0.057, *p* = 0.54 for OS and *r*^2^ = 0.0007, *p* = 0.94 for TTNT) (Figure 4).

### 3.3. Bulky Disease

All six patients presenting with bulky disease (maximum tumor diameter >7 cm) achieved a CR at three-month ^18^F-FDG-PET/CT. Five (82%) maintained CR at a subsequent ^18^F-FDG-PET/CT performed at 12 months. The other patient elected not to be reimaged but remained in clinical remission. Two (33%) experienced relapse at 30 and 42 months, respectively, and required retreatment. All six patients (100%) remain alive at the time of analysis. No significant relationship was identified between the presence of disease bulk >7 cm and TMTV.

### 3.4.Transformation

Two patients in the study experienced disease relapse with transformation. Both patients failed to achieve a CR at three-month ^18^F-FDG-PET/CT and both subsequently succumbed to their disease following failure of salvage therapy. Neither patient had bulky disease at diagnosis and their respective baseline TMTV were 72 and 250 cm^3^, respectively.

## 4. Discussion

Imaging of early response in the management of cancer is particularly important in FL. Given that the majority of patients will need no further therapy for over a decade. The median OS of FL now approaches 15 years [25], but there is an important subpopulation of up to 20% who relapse early and require close monitoring and repeated treatment [3]. Early identification of this poor prognosis subset of FL is of critical importance to optimize personalized therapeutic intervention. We have shown in our prospective series of first-line RIT FL with ^131^I-rituximab that ^18^F-FDG-PET/CT at three months after induction therapy reliably predicts outcome with respect to TTNT. Failure to obtain a CR on Deauville criteria defined the 10–20% of FL patients who will require close monitoring and probable early re-treatment. In our study, the 88% who achieved CR at three months could be reassured that it would be very unlikely that they will required further treatment within 10 years. This individual patient reassurance adds greatly to their quality of life and minimizes the use of costly health resources.

In our, admittedly small, cohort, without high tumor burden, TMTV on the baseline ^18^F-FDG-PET/CT imaging study did not stratify FL for outcomes with statistically significant reliability. Furthermore, no significant correlation was demonstrated between baseline TMTV values and time dependent end-points. Whilst TMTV has been shown to have prognostic utility in high-tumor-burden FL [3], the same remains to be demonstrated in the setting of RIT for advanced FL. Clearly, our cohort in whom less than 10% had baseline high-tumor-burden (7% and 9%, respectively, by TMTV ≥510 cm^3^ and nodal mass >7 cm in diameter) is not comparable to those analyzed in Meignan and colleagues landmark publication [3]. Furthermore, it can reasonably be argued that the difference in therapy may have affected the prognostic value of the baseline measure. However, despite its potential prognostic utility in FL, TMTV still remains prone to observed differences in quantitative data by different PET systems and measurement error [26]. Finally, the major issue for TMTV measurement in FL is the choice of method, as accepted by Meignan, the 41% SUV_max_ methodology is the best “compromise” to measure lesions of varying size, SUV^max^ and SUV_max_/background ratio [27]. Despite this, given the demonstrated low interobserver variability and with an expanding therapeutic arsenal, it is reasonable for ongoing studies of FL to analyze the utility of baseline TMTV in the setting of novel therapies.

Over the last five years, there has been a concerted effort to standardize imaging response assessment in lymphoma. The initial 2007 International Harmonization Project tumor response classifications have been updated, first with the 2009 Deauville 5-point scale (D5PS) and more recently by the 2014 Lugano Criteria, which incorporates the D5PS [28]. This standardization has allowed for unified, comparable response assessment and lead to numerous studies, across various lymphoma subtypes (including Hodgkin lymphoma), firmly establishing the prognostic importance of post treatment ^18^F-FDG-PET/CT response and allowing for response adaptive therapies [29].

It must be noted that, in our cohort, the significance of post treatment imaging response was not demonstrated with respect to overall survival. This finding is in keeping with published results from analyses of FL patients in the PRIMA [30] and FOLL05 [31], in whom PET positivity within three months of completing therapy was only able to accurately identify those patients at high risk of progression and was a more powerful prognostic indicator than FLIPI [32]. Studies in which PET has robustly predicted for a significant difference in OS have often not included patients who have received maintenance rituximab therapy [10,32], which is now an established standard of care. Our cohort were all assigned to maintenance rituximab, and this difference in the significance of post treatment ^18^F-FDG-PET/CT with respect to TTNT and OS may be accounted for by the beneficial effect and markedly improved OS seen in the rituximab era. However, the issues of the true prognostic role of ^18^FDG-PET/CT remain to be addressed, as we are yet to improve the survival of the high-risk cohort that fail to achieve a CR following therapy.

Non-imaging prognostic indices such as FLIPI and FLIPI2, monocytes and lymphocyte counts did not reliably predict survival outcome in our patients. However, such routine screening tests are significantly less expensive than PET/CT imaging studies. Moreover, baseline monocytosis and or FLIPI were able to predict TTNT, in our study, with *p*-values of 0.03 and 0.01, respectively.

Finally, in the last decade, there have been major advances in the application and analysis of the prognostic impact of tumor genetic profiles. The m7-FLIPI, which incorporates mutation analysis of seven genes (*EZH2*, *ARID1A*, *MEF2B*, *EP300*, *FOXO1*, *CREBBP*, and *CARD11*) combined with Eastern Cooperative Oncology Group (ECOG) status, has recently been validated as a clinicogenetic risk model. In the validation cohort, m7-FLIPI was able to stratify patients that have high and low risk with five-year failure-free survival of 25% (95% CI 12·50–49·99) and 68% (95% CI 58·84–79·15), respectively [5]. More importantly, it has led to the identification of mutated EZH2 as a protective factor in FL. Genes have been incorporated into the panel based on their prognostic weight, without consideration for their biologic function. This method avoids a priori selection but is contrary to traditional methods of only considering clinically relevant variables. Undoubtedly, the future of FL prognostication will involve a “bioscore”, but, for the time being, high throughput genetic analysis remains accessible only in large well-funded centers [33]. Furthermore, genetic stratification is currently a baseline assessment. It does not yet obviate the need for adaptive therapeutic strategies, which predominantly rely upon imaging response.

We conclude that imaging of early response after induction therapy of FL, applying standard Deauville criteria to the three-month ^18^F-FDG-PET/CT, is a robust, accessible and reliable prognostic indicator of TTNT in the individual patient.

Prognostication involves looking into the future. Existing paradigms only take into account two time points in a patient journey, diagnosis and subsequent response to therapy, either in the form of an interim or end of treatment analysis. Within each, the three major pillars used to inform our decision-making are patient-related factors, tumor biology and imaging. In the traditional paradigm, at diagnosis, emphasis is placed upon patient and tumor-related factors, with imaging used predominantly to guide biopsies and to diagnose and stage the disease. Imaging gains importance during follow-up, as it is the single most important methodology for monitoring disease response to therapy.

Our understanding and analysis of tumor biology now allows us to better define the small subset of FL patients likely to relapse. Thus, in future, we can conceptually concentrate our molecular imaging resources to closely monitor this cohort and complement the PET scan with molecular tumor volume estimation and baseline genetic stratification, which may potentially predict tumor transformation.

## 5. Conclusions

Our analysis of 10-year follow-up of the Phase II prospective INITIAL Study shows that early ^18^F-FDG-PET/CT imaging assessment of response, on Deauville criteria, is a predictor of time-to-next-treatment for first-line ^131^I-rituximab RIT of advanced follicular lymphoma. In a disease whose median failure-free-survival is in excess of 10 years, our study supports the utility of post-treatment imaging for the early identification of higher risk subjects and may facilitate future adaptive therapeutic strategies.

## Figures and Tables

**Table 1:** Patient characteristics.

	*n*	%
Total patients	68	
Male	31	46
Female	37	54
Age	-	-
<60	36	53
≥60	32	47
Disease Stage		
I/II	17	25
III/IV	51	75
*TMTV*		
<510 cm^3^	55	81
≥510 cm^3^	5	7
ND	8	12
Bone marrow involvement		
Yes	18	26
No	50	74
Bulky disease (>7 cm)		
Yes	6	9
No	62	91
Serum LDH		
Normal	57	84
Elevated	11	16
B2-microglobulin		
Normal	58	85
Elevated	8	12
ND	2	3

TMTV: Total-metabolic-tumor volume, ND: not done, LDH: Leukocyte dehydrogenase.

**Table 2:** Survival risk ratios by prognostic factor.

Prognostic Factor	RR	CI	*p*-Value
Age ≥ 60 year	4.3	1.2–14.8	0.04
Gender F:M	1.1	0.3–3.9	0.9
Lymphocyte count ≥ 1.2 × 10^6^/L	1.5	0.4–5.4	0.6
Monocyte count > 0.8 × 10^6^/L	0.8	0.1–5.0	0.8
Bulky disease (>7 cm)	3.0	0.3–30.1	0.3
FLIPI > 3	3.3	1–12.5	0.09
PR/SD/PD at 3 month PET/CT (Deauville > 3)	3.2	0.47–21.32	0.08
Baseline TMTV > 510 cm^3^	1.2	0.1–11.5	0.8

FLIPI: Follicular lymphoma international prognostic index, PR: partial remission, SD: stable disease, PD: progressive disease, TMTV: Total metabolic tumor volume, RR: Risk ratio, CI: Confidence interval, PET/CT: fluorine-18-fluorodeoxyglucose positron emission tomography with computed tomography.

**Table 3:** Time-to-next-treatment risk ratios by prognostic factor.

Prognostic Factor	RR	95% CI	*p*-Value
Age ≥ 60 year	1.4	0.4–4.4	0.7
Gender F:M	1.3	0.4–3.9	0.6
Lymphocyte count ≥ 1.2 × 10^6^/L	2.1	0.7–6.4	0.2
Monocyte count > 0.8 × 10^6^/L	9.2	1.5–55.8	0.03
Bulky disease (>7 cm)	2.1	0.3–13.8	0.7
FLIPI ≥ 3	5.6	1.8–17.5	0.01
PR/SD/PD at 3 month PET/CT (Deauville > 3)	7.4	1.0–56.3	<0.0001
Baseline TMTV > 510 cm^3^	1.1	0.1–9.9	0.8

FLIPI: Follicular lymphoma international prognostic index, PR: partial remission, SD: stable disease, PD: progressive disease, TMTV: Total metabolic tumor volume.
